# Mental health of children in ukraine. risks and challenges today

**DOI:** 10.1192/j.eurpsy.2024.537

**Published:** 2024-08-27

**Authors:** M. Markova, T. Aliieva, O. Piontkovska

**Affiliations:** Kharkiv National Medical University, Kharkiv, Ukraine

## Abstract

**Introduction:**

Today, the most vulnerable group of Ukrainians are children. Their physical and mental health has been tested since 2020, from COVID-19 to 2022, the start of a full-scale-russia’s war against Ukraine. The children of Ukraine were the first to feel the changes, since a complete change in life and the principles of acquiring primary adaptive skills of social interaction were distorted by COVID-19. Subsequently, new challenges in the form of war deformed the idea of life, happiness and the future.

**Objectives:**

Studying the level of adaptation potential in children and adolescents living in the front-line zone in Ukraine.

**Methods:**

The examination included the use of clinical-psychological, psychodiagnostic and psychometric research methods.

**Results:**

The study in 2021 involved 217 children and adolescents with signs of maladjustment. In 2022, 378 children and adolescents with signs of maladjustment, of which 285 children are still in the frontline zone of Ukraine, 93 children, at the time of 2022, were taken abroad and returned to Kharkov in 2023.

During the initial analysis of the results, it was revealed that children with low adaptive resources are more susceptible to showing signs of maladjustment. One of the main factors of an adaptation resource is interaction with others (direct communication). It was this criterion that became the primary frustrating factor for children in the first months of the war. External isolation has led to a lack of communication between children and everyone in Ukraine, who during COVID-19 have adapted to a way of communicating while staying at home in physical isolation. War is a powerful independent psychogenic factor for the formation of maladaptation, but in today’s realities it has also become an additional trigger for a previous psychogenic event in the life of Ukrainians.

Parents, for their part, note a sharp deterioration in the well-being of their children, frequent complaints of headaches (89.4%), overwork, aggressiveness (81,5%), closed-mindedness (78,6%), health complaints (74,5%), which is why they have to often consult a doctor and endlessly carry out diagnostics in search of reasons for deterioration (72,3%), weight loss (64,5%). The data indicate the frustration of being in the front-line zone, which aggravates mental health and triggers the process of disruption of adaptation and mental health of children and adolescents.

**Conclusions:**

The study is aimed at developing a psychorehabilitation program for children and adolescents with a low level of adaptive resource.

**Disclosure of Interest:**

None Declared

